# Left ventricular strain distribution in healthy dogs and in dogs with tachycardia-induced dilated cardiomyopathy

**DOI:** 10.1186/1476-7120-11-43

**Published:** 2013-12-05

**Authors:** Kenya Kusunose, Youhua Zhang, Todor N Mazgalev, James D Thomas, Zoran B Popović

**Affiliations:** 1 Department of Cardiovascular Medicine, Cleveland Clinic, Heart and Vascular Institute, 9500 Euclid Avenue, Desk J1-5, Cleveland, OH 44195, USA; 2 Department of Molecular Cardiology, Cleveland Clinic, Heart and Vascular Institute, Cleveland, OH 44195, USA

**Keywords:** Strain echocardiography, Twist, Tachycardia induced cardiomyopathy

## Abstract

**Background:**

Recently, left ventricular (LV) strain distribution pattern has been assessed in several cardiac disease states. Tachycardia-induced cardiomyopathy (TIC) is an animal model of non-ischemic cardiomyopathy well characterized in terms of global LV dysfunction but with poor understanding of regional variability in LV function. We hypothesized that TIC induces specific changes in LV strain distribution pattern.

**Methods:**

Twenty five adult mongrel conscious dogs were trained to lie down calmly for echocardiography. In seven selected dogs, we implanted pacing system for TIC induction under general anesthesia. We measured LV geometry and function, strains, and torsion before and after the development of TIC in awake non-sedated state.

**Results:**

In 25 healthy dogs, all three types of normal strain significantly increased from base to apex (p <0.05), while a definite and recognizable twist could be measured due to presence of shear strain. In 7 dogs with TIC, marked changes in LV mechanics occurred throughout the cardiac cycle, resulting in decrease of strain (p <0.001), twist (p <0.05), and negative peak twist rate (p <0.05). Interestingly, the relative decrease of strain due to TIC was more pronounced in the apex (p < 0.001), with the radial strain decreasing the most (p < 0.05).

**Conclusion:**

TIC is accompanied by decreased systolic LV strain and twist deformation, as well as loss of early diastolic recoil. In addition, the decrease of strain was more profound in the apex. This “reverse” distribution of LV strain may help us understand LV dysfunction in the presence of nonischemic etiology.

## Background

Left ventricular (LV) global and regional wall deformation can best be described by the assessment of normal and shear strains. Speckle tracking echocardiography (STE) emerged as powerful noninvasive method to quantitate the three components of normal strain: circumferential (S_C_), longitudinal (S_L_) and radial (S_R_) [1]. In addition, STE can quantitate torsional deformation of LV that is a consequence of shear strain occurring in the longitudinal-circumferential plane and caused by the oblique orientation of subepicardial myofibers. LV torsional deformation parameters that are most frequently measured are LV twist (a difference in rotation between basal and apical part of the left ventricle) and LV twisting and untwisting velocities [2].

Quantitation of myocardial deformation is an emerging field of clinical cardiac imaging. In addition, recent clinical works using STE shows that there are significant differences in regional strain in several cardiomyopathies even in the absence of ischemia. Knowledge of characteristic LV strain distribution pattern facilitates diagnosis for constrictive pericarditis [3], cardiac amyloidosis [4] and hypertrophic cardiomyopathy [5]. However, in individual patients additional or competing factors may influence the strain distribution [6]. The characteristic strain distribution maps from homogeneous models of health and disease may help in differential diagnosis of individual patients.

In this paper we use STE to simultaneously assess all three components of normal strains as well as torsional deformation parameters in healthy dogs and in dogs that developed tachycardia-induced cardiomyopathy (TIC). Thus the aim of this study was 1) to define the range of normal values of LV strain components and torsional deformation in healthy mongrel dogs, 2) to define absolute and relative changes in myocardial deformation after the development of TIC, and 3) to define the strain distribution in healthy and TIC dogs as the strain distribution maps may help in differential diagnosis of TIC in clinical setting.

## Methods

### Study population

All the animal experiments reported in this article were approved by the Institutional Animal Care and Use Committee of Cleveland Clinic and were in compliance with the National Institutes of Health Guide for the Care and Use of Laboratory Animals.

### Procedures

We analyzed the echocardiographic data of a total of 25 adult healthy mongrel dogs (both sexes, body weight 21 to 35 kg). All echocardiography data acquisition was planned and performed prospectively. In addition, 7 adult healthy mongrel dogs (both sexes, body weight 22 to 27 kg) were studied again after induction of TIC. Briefly, these 7 dogs were premedicated with thiopental sodium (20 mg/kg iv.), intubated and mechanically ventilated by a respirator with room air supplemented with oxygen. Anesthesia was then maintained with 1–2% isoflurane throughout the experiment. A custom, high-rate ventricular pacemaker (St. Jude Medical) was connected to right ventricular pacing lead implanted in the right ventricular apex [7]. Two weeks after device implantation, the ventricular pacemaker was turned on to pace the ventricles at 220 bpm for 4 weeks to induce heart failure. The development of left ventricular dilatation was confirmed by echocardiography. The animal status was carefully monitored on a daily basis. The animals contributed to our prior experiments [7,8].

### Data collection

Echocardiography was performed using Vivid 7 echocardiography machine (GE Medical, Milwaukee, WI, USA). Dogs were trained to lie down calmly on their side and were imaged in left decubitus while awake. In TIC dogs, the pacemaker was turned off and echocardiographic data was acquired after a stabilization period of >15 minutes. Two-dimensional echocardiography data were collected using a dual harmonic 1.7/3.4 MHz or 2.0/4.2 MHz sector transducer. The minimal frame rates acquired during standard two-dimensional echocardiography were 50 frames s^-1^. Data were digitized and stored in a proprietary format for further analysis. Apical 4-chamber, 2-chamber, long axis, and parasternal short axis views (at LV base, mid and apex) were obtained.

### Data analysis

Data were analyzed using EchoPAC PC (GE Medical Systems, Milwaukee, WI). LV end-diastolic and end-systolic volumes were measured from the 4-chamber and 2-chamber views by the Simpson equation. We used a speckle-tracking algorithm incorporated into EchoPAC PC performed by a trained observer [9]. The region of interest was overlaid on a cross section of the ventricular silhouette at the image corresponding to the minimal endocardial area. The software algorithm then automatically divided the LV apex view and LV short-axis view into six segments and three levels for speckle tracking throughout the cardiac cycle. The tracking quality was then visually inspected, and, if it was satisfactory for at least five segments, the tracing was accepted. Segmental S_L_, S_C_, and S_R_ curves were then constructed to analyze regional strains and averaged to obtain global strain curves. End-systolic strain data were analyzed. Apical and basal rotations were determined from the respective 2D short-axis images. LV twist was computed as the difference between apical and basal rotation, while considering counterclockwise rotation as a positive value and clockwise as negative. LV twisting and untwisting rate was computed as the peak systolic and diastolic time derivative of twist, respectively. For the analysis of all data, at least three heart beats were measured. The mean value was used for statistical analysis.

### Statistical analysis

Statistical analysis was performed using a standard statistical software package (SPSS software 14.0, SPSS Inc.). Continuous data are presented as mean ± SD. Comparisons between pre and post TIC data were performed by paired t test in the 7 dogs. To assess the presence of regional strain variability, we used a repeated-measures analysis of covariance with LV walls as a fixed factor, animal number as random factor, and level codes (0 = basal, 1 = mid and 2 = apex) as a covariate. Analysis of simple contrasts was used to assess the differences between individual walls. To compare relative change in various strain components after TIC was induced we first expressed change in segmental strains as percent change. We then performed a two-way repeated measures analysis of covariance with type of strain (circumferential, radial and longitudinal) as fixed factor, animal number as random factor, and level codes (0 = basal, 1 = mid and 2 = apex) as a covariate. Analysis of simple contrasts was used to assess the differences between individual strains. Inter- and intra-observer variability was examined for the three components of LV strain (S_L_, S_C_ and S_R_). Measurements were performed in a group of 14 subjects (pre and post TIC data in the 7 dogs) by one observer then repeated on two separate days by two observers who were unaware of the others’ measurements and of the study time point. Reproducibility was expressed as the mean percentage error (absolute difference divided by the average of the two observations). Statistical significance was defined by p < 0.05.

## Results

### LV deformation in healthy dogs

**Figure 1:**
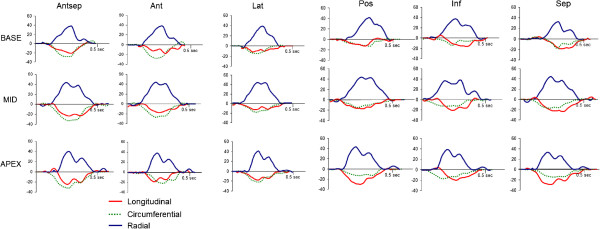
An example of segmental longitudinal, circumferential and radial myocardial strain profiles obtained during a single cardiac cycle in a healthy dog (A).

**Figure 2:**
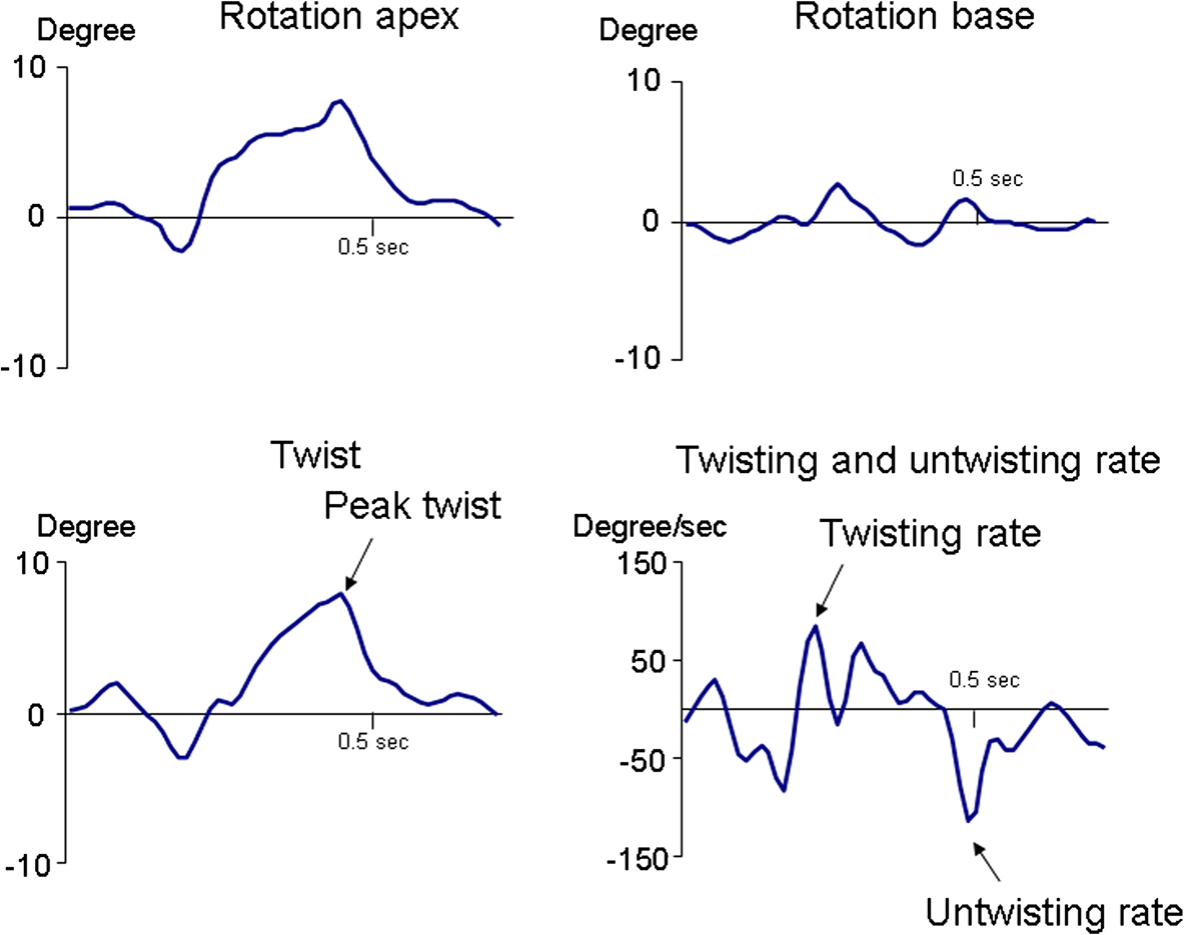
An individual example of rotation, twist and twisting velocity profiles obtained in a healthy dog.

Figure 1 shows characteristic S_L_, S_C_, and S_R_ profiles of individual segments obtained in a healthy dog. Figure 2 shows apical and basal rotation and corresponding twist and twist velocities from the same dog. Table 1 summarizes global LV mechanics in all 25 dogs studied.

**Table 1:** Global LV mechanics in healthy dogs (n = 25)

	**Baseline**
Heart rate (bpm)	123 ± 26
LV end-diastolic volume (ml)	52 ± 8
LV end-systolic volume (ml)	20 ± 6
LVEF (%)	61 ± 8
Global longitudinal strain (%)	-18 ± 4
Global circumferential strain (%)	-17 ± 4
Global radial strain (%)	39 ± 20
Rotation (degree)	
Base	-3.2 ± 1.4
Mid	2.1 ± 1.3
Apex	4.4 ± 1.5
Peak twist (degree)	8.1 ± 4.4
Positive peak twisting rate (degree/sec)	115 ± 49
Negative peak twisting rate (degree/sec)	-121 ± 47

**Figure 3:**
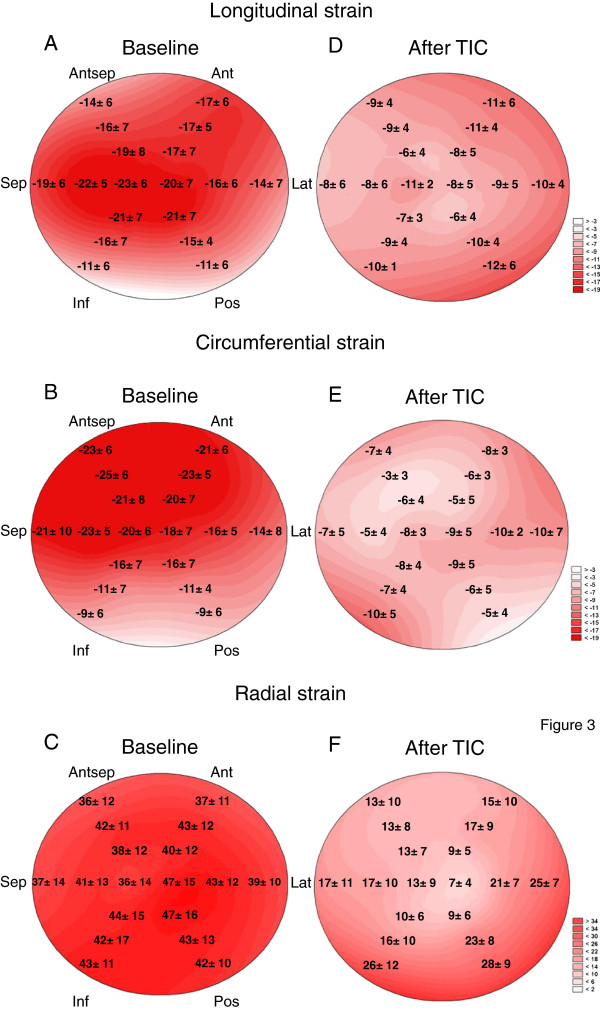
**Segmental end-systolic strains of dogs at baseline (A-C) and dogs with tachycardia induced cardiomyopathy (TIC) (D-F).** Antsep; antero-septal wall, Ant; anterior wall, Lat; antero-lateral wall, Post; posterior wall, Inf; inferior wall, Sep, infero-septal wall.

Segmental S_L_, S_C_, and S_R_ are presented at Figure 3A-C. There was a significant increase of absolute values of S_L_ in base to apex direction (p <0.001), with absolute averaged S_L_ increase of 1.5% per each level. There was also a significant difference between individual walls (p =0.017), with septal wall having highest absolute value of S_L_ (p <0.05) for septum vs. every other individual wall). Similarly, there was a significant increase of absolute value of S_C_ in base to apex direction (p =0.005), with absolute averaged S_C_ increase of 1.1% per each level. There was also significant difference between individual walls (p <0.001), with septal wall having higher absolute value of strain compared to posterior, lateral, or inferior walls (p <0.05 for septum vs. posterior, lateral, or inferior walls). Finally, there was a significant increase of S_R_ in base to apex direction (p =0.035), with absolute averaged S_R_ increase of 2.1% per each level. There were no differences between individual walls.

In summary, in normal dogs there is significant segmental strain variability, with absolute values of strain uniformly higher in the apex, and with septum having higher values of S_C_ and S_L_.

### LV deformation in dogs with TIC

**Figure 4:**
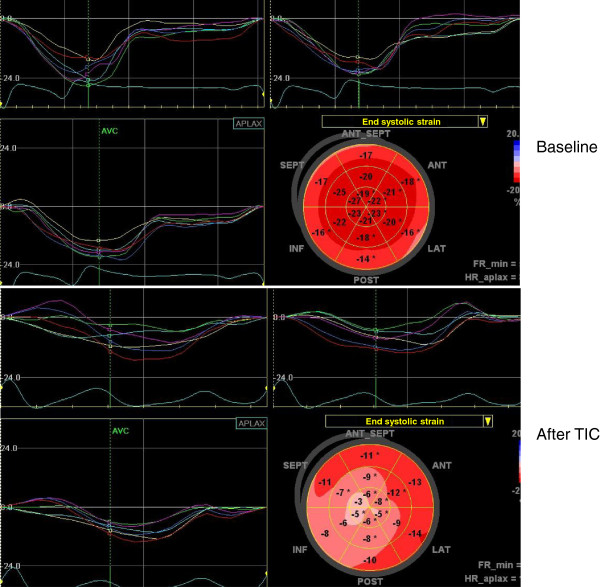
The representative longitudinal strain curves in a tachycardia induced cardiomyopathy (TIC) dog.

Figure 4 shows a representative case for S_L_ in a TIC dog. Table 2 summarizes global LV mechanics of 7 dogs with TIC, at baseline and during heart failure. With TIC a dramatic worsening of ejection fraction and increase of LV volumes occurred, accompanied by more than 50% decrease in S_L_, S_C_, and S_R_. Changes also occurred in distribution of normal strain. Thus, after TIC, there was a significant decrease of absolute value of S_L_ in base to apex direction (p =0.015), with absolute strain decrease of 1.1% with each level, and no detectable difference between individual walls detected (Figure 3D). Segmental distribution of S_C_ after TIC showed an absence of any apex-to-base gradient in circumferential strains, with significant difference between segments (p =0.04) (Figure 3E). A significant decrease of S_R_ in base to apex direction (p <0.001) was observed, with absolute strain decrease of 5.3% with each level. There was no difference between individual walls (Figure 3F). Finally, TIC lead to decreased absolute values of mid and apical rotation, as well as of twist and untwisting rates (Table 2).

**Table 2:** Global LV mechanics of dogs (n = 7) with tachycardia induced cardiomyopathy (TIC) at baseline and during cardiomyopathy

	**Baseline**	**TIC**	**p value**
Heart rate (bpm)	124 ± 25	159 ± 25	0.009
LV end-diastolic volume	56 ± 3	83 ± 12	<0.001
LV end-systolic volume	22 ± 5	66 ± 9	<0.001
LVEF (%)	60 ± 9	21 ± 5	<0.001
Global longitudinal strain (%)	-19 ± 5	-9 ± 5	<0.001
Global circumferential strain (%)	-17 ± 7	-7 ± 5	<0.001
Global radial strain (%)	41 ± 19	16 ± 14	<0.001
Rotation (degree)			
Base	-3.0 ± 1.4	-2.1 ± 1.7	0.21
Mid	1.9 ± 1.2	-1.7 ± 1.2	0.002
Apex	4.3 ± 0.9	1.2 ± 0.6	<0.001
Peak twist (degree)	8.9 ± 5.7	2.4 ± 2.1	0.03
Positive peak twisting rate (degree/sec)	115 ± 67	58 ± 22	0.11
Negative peak twisting rate (degree/sec)	-145 ± 69	-62 ± 35	0.04

**Figure 5:**
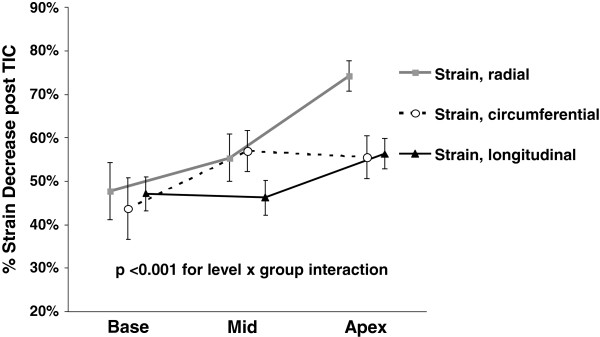
**The relative decrease of longitudinal, circumferential, and radial end-systolic strains after induction of tachycardia induced cardiomyopathy (TIC) at base, middle, and apex of the left ventricle.** All strains decreased significantly, with the decrease of strains significantly more pronounced as one moved from the base towards the apex (p <0.001). Relative decrease significantly varied between various strains (p < 0.05) and was most pronounced for radial strain. Error bars represent SEM. TIC: tachycardia induced cardiomyopathy.

Figure 5 summarized the relative changes in normal strains occurring with TIC. The relative decrease in all normal strains increased while going from the base to apex (p <0.0001). S_L_, S_C_, and S_R_ decreased to different extents (p =0.048). The decrease was more prominent for S_R_ than for S_L_ (p =0.02), while the difference between S_R_ and S_C_ was borderline (p =0.07).

In summary, induction of TIC led to decrease of all parameters of LV deformation. The decrease was more prominent in the apical parts of the ventricle, and was the most prominent with S_R_. The intra- and inter-observer variability of the S_L_ were 6.8 ± 5.2% and 7.2 ± 6.1%, S_C_ were 8.2 ± 5.5% and 10.1 ± 6.6%, and S_R_ were 8.3 ± 6.8% and 11.2 ± 7.5%.

## Discussion

In this paper, we define the normal values of LV strain components and their segmental distribution using speckle tracking echocardiography in a large group of healthy, conscious, mongrel dogs. We show here that the average values of strain in dogs are similar to human population. Moreover, similar to healthy human population, all three types of normal strains significantly increased from base to apex, while a definite and recognizable twist could be measured due to presence of shear strain [10]. With TIC, marked changes in LV mechanics occurred, resulting in dramatic decrease of linear strain and twist. Interestingly, the decrease of strain during TIC was more profound in the apex, with the radial strain decreasing the most. This “reverse” distribution of LV strain may help us understand LV dysfunction in the presence of nonischemic etiology. In addition, while previous studies have quantified strains in healthy dogs, the significance of these studies was often limited: strains were quantitated with tissue Doppler imaging (angle-dependent), sedation or anesthesia were used, one particular strain component was measured, or non-specific dog breeds were studied. This precluded generalizability of the data [11-14].

### Myocardial deformation pattern: human studies

Three-dimensional myocardial deformation of human myocardium was characterized by a number of independent techniques. Ingels et al. originally implanted mid-myocardial markers into transplanted hearts to show the mean values of S_L_, S_C_ and twist to be 12.5%, 13.5% and 18° post transplantation, respectively [15]. Moore et al. used 3-dimensional tagged magnetic resonance imaging [16] of healthy volunteers to show slightly higher values mean values of S_L_, and S_C_ of 17% and 20%, respectively, a mean wall thickening (an S_R_ analogue) of approximately 60%, and a mean twist of 12.3°. More importantly, they showed a consistent increase of deformation from the base towards the apex in all strain components, with apex-to-base differences in S_L_, S_C,_ and wall thickening of 4%, 8%, of 22%, respectively. Finally, healthy volunteers studies using STE confirmed base to apex increase in myocardial deformation for both S_L_ (from -17% to -20.2%) [10] and S_C_ (from 19.6 to 24.5%) [17], with a twist of 9.7° [17]. In summary, human myocardium shows a deformational pattern characterized by normal strain components increasing from base to apex, with LV twist between 10 and 18°. These patterns are clinically relevant, as loss or accentuation of base-to apex gradient of LV deformation is associated with specific disease states [4,18].

### Prior studies in healthy dogs

While multiple previous studies attempted to define normal values of myocardial deformation in dogs, they are often limited due to their focus on a non-specific dog breed, or by color tissue Doppler imaging (angle-dependent) [19,20]. Several recent papers attempted to define normal strain values using the modern technique of STI. In a group of dogs of varying breeds ranging in size from 2 to 52 kg and studied while non-sedated, Chetboul et al. obtained mean S_R_ of 47 ± 12% at papillary muscle level [21], and mean LV twist of 8 ± 4° [22]. Takano et al. showed that anesthetized healthy beagle dogs have S_C_ at papillary muscle level of 20 ± 4%. While these results were similar to ours, another study of awake mongrel dogs imaged in a 4 chamber view showed S_L_ value of only -16 ± 5% [23]. While the results of these studies, except [23], are mostly similar to our findings, they are all limited as they either use of a single view, analyze of a single strain component, study a specific breed, or use of anesthetic agents.

### Transition to heart failure phenotype

We have already shown that 4 weeks of pacing leads to LV dysfunction associated with marked neuro-hormonal activation [7]. We here show that this is further associated with a dramatic decrease of all normal strain components and LV twist. This decrease was substantially larger than the one observed in a recent study of anesthetized dogs with TIC, possibly reflecting the blunting effect of anesthetics [14]. Furthermore, decrease in strains was more prominent at the LV apex, with S_R_ showing the largest relative decrease. Interestingly, a recent small clinical study of patients with dilated cardiomyopathy [24] showed that a multiparametric strain Z-score, a marker of 3-dimensional LV deformation, is significantly smaller at LV apex. In addition, there was a loss of apex-to base gradient of circumferential strains in TIC. One possible explanation is that the initial decrement of ventricular contraction affects the longitudinal axis, whereas circumferential axis is relatively preserved in the early phase regardless of the physiopathological model. These findings, taken together, indicate that a decrease of myocardial contractility seen in dilated cardiomyopathy has larger effect on strains in apical segments. This may be puzzling, since tachycardia as pathophysiologic mechanism should have similar impact in all LV regions. A plausible explanation for a larger increase in LV stress in the apex could be that basal and mid-ventricular parts were protected from stress increases and subsequent remodeling by the mitral annulus and papillary muscles [25].

### Clinical implications and limitations

The frequency of TIC is often under-appreciated. TIC may be present in the setting of atrial fibrillation, atrial flutter, or any other chronic atrial tachycardia, as well as in the setting of frequent premature ventricular contractions [26,27]. The clinically relevant issue is that it can be present even after termination or interruption of tachycardia [26,27]. The strain distribution pattern shown in this paper may help differentiate that type of cardiomyopathy from other possible causes of decreased systolic function.

On the other hand, the exact clinical counterpart of the high-rate ventricular pacing model used in our study is rare. However, clinical practice, a drop in ejection fraction can be seen with automatic atrial tachycardia; a recent paper has shown that, if paced to the similar heart rate, both right atrial pacing (a surrogate of atrial tachycardia) and RV pacing produce a similar drop in EF [28]. Similarly, we have shown that in atrial fibrillation model of chronic TIC, when dogs with initially RV-pacing induced TIC are subjected to chronic atrial fibrillation with rapid ventricular response they maintain essentially the same severity of LV dysfunction and dilatation [8]. Thus, it is likely that all three types of TIC (that is, regular ventricular pacing, regular atrial pacing, and rapid irregular supraventricular rhythm of atrial fibrillation) lead to similar severity and pattern of cardiac dysfunction if the hearts are subjected to the similar degree of tachycardia. However, more studies are needed to clarify this issue.

RV pacing may in itself worsen LV function through induction of dyssynchrony, or by causing changes in myocardial histology. However, the effects of RV pacing on dyssynchrony are very transient, and its hemodynamic effects often resolve within few beats [29]. On the other hand we may not definitely rule out some impact of RV pacing-induced myocardial histology changes on LV function. However, the duration of RV pacing was relatively brief at 4 weeks and there was no significant difference between the apical septal (near pacing site) and basal lateral LV wall thickness (6 ± 2 vs 7 ± 2 mm, p = 0.26). Previous studies showed no difference in the amount of cardiac dysfunction between animals that were atrially or ventricular paced at the similar heart rate [28]. Furthermore, stopping ventricular pacing resulted in normalized LV ejection fraction and neurohormonal profiles [30].

Finally, when compared with the “gold-standard” derivation of strains from tracking of implanted radio-opaque markers or tagged magnetic resonance imaging, two dimensional STE has limitations, as there is a possibility that 2D strains fail to account for the complex deformation of the LV.

## Conclusion

In conclusion, we defined normal values of global and segmental LV deformation in a large series of awake mongrel dogs. We also quantitate the global and segmental impact of TIC on LV deformation. Finally, we identified the characteristic pattern of a decrease of LV function induced by TIC, with its most profound effect in LV apex. These findings may help accurate detection of abnormalities of LV contraction in dogs, and elucidate the pathophysiology of LV contraction in tachycardia-induced cardiomyopathy.

## Competing interests

All authors declare that they have no competing interests concerning this study.

## Authors’ contributions

All authors participated in the initiation and design of the study. All authors participated in the experiments and data collection. All authors participated in data analysis and interpretation of the results. All authors read and approved the final manuscript.
